# Atlas of External Diseases of the Eye

**Published:** 1900-12

**Authors:** 


**Atlas of External Diseases of the Eye.** By A. Maitland Ramsay, M.D. Pp. xvi., 195. Glasgow: James MacLehose & Sons. 1898.

The plates in this atlas are nearly all executed from photographs taken under the personal superintendence of the author, from actual cases under his care at the Glasgow Eye Infirmary.

Both the photogravures and the chromo-lithograph plates are admirable in execution, and illustrate faithfully a large number of the most characteristic affections of the eyes and eyelids. Some are shown in association with the more general conditions typical of the disease of which they form a part. The representations of "Interstitial Keratitis," "Strumous Ophthalmia," "Tumours of the Sphenoid," and "Aneurism of the Orbit," among others, are very accurate as well as artistic.

The book does credit to its author and to its publisher—

it is worthy to take rank with the atlases of Dalrymple, of Ruete, and of Demours.

Besides the excellence of the illustrations is that of the general arrangement of the book. Each chapter is headed by the Greek derivation of the name of the disease, and by the synonyms by which it is known in different countries. The descriptions are simple, but accurate and definite, and it may be fairly claimed that they assist in making the book "useful to medical men in general practice, who may not have many opportunities of visiting the wards and clinique of an ophthalmic institution."

Almost all the well-recognised external eye diseases are adequately dealt with, and when the special ætiological factor is a bacillus the particular organism is indicated if not described. Spring catarrh is not illustrated and is too briefly described, and this could no doubt be said of some other affections; but occasional defects do not spoil the general excellence of the work—which will be a valuable book of reference to the general practitioner, a real addition to the library of the ophthalmic surgeon, and worthy of a place on the shelves of any book collector.

